# Supplementary material for: Virtual care use during the COVID-19 pandemic and its impact on healthcare utilization in patients with chronic disease: A population-based repeated cross-sectional study
Source: PLoS One. 2022 Apr 25;17(4):e0267218. doi: 10.1371/journal.pone.0267218 (PMC9037937; doi:10.1371/journal.pone.0267218)

Supplement 1

The following billing codes were included:

- Prior to April 1st: 2020: premium fee codes B100A, B200A, B101A, B201A, B102A, B202A; Tracking fee code: B099A
- After April 1st, 2020: B103A, B203A
- After March 14th, 2020: (temporary OHIP fee codes due to COVID-19) K080, K081, K082, K083.

Patient clinical subgroups:

1. Patients with **COPD** [COPD]:

- Record in the ICES COPD database any time prior to index virtual visit

1. Patients with **heart failure** [CHF]:

- Record in ICES CHF database any time prior to index virtual visit

1. Patients with **asthma** [ASTHMA]:

- Record in ICES ASTHMA database any time prior to index virtual visit

1. Patients with **hypertension** [HYPER]:

- Record in the ICES HYPER database any time prior to index virtual visit

1. Patients with **angina** [NACRS, OHIP]:

- At least one ED visit within 12 months prior to index virtual visit with any of the following codes:
  ICD-9: 411.1, 413.0, 413.1, 413.9, 786.51, 786.52

ICD-10: R07.1–R07.4, I20.0, I20.1, I20.8, I20.88, or I20.9

1. Patients with **diabetes** [ODD]:

- Record in the ICES ODD database any time prior to index virtual visit

1. Patients with **mental health** service use in the 3 years prior to the index virtual visit. Please identify patients with any OHIP claim that lists any one of the following location codes: O,L,H,P, (exclude all G codes) along with at least one of the OHIP feecodes listed below. 1 visit is counted as 1 claim/IKN/physnum/servdate.

- Primary Care Provider, PCP (SPEC=00): General service code (A001, A003-A008, A888, A901, A905, K080, K081, K082, K083, B099, B100, B200, B101, B201,B102, B202, B103, B203, B209) AND with a mental health diagnostic code (295-298, 300-304, 309, 311)

OR

- Mental health service code (K005, K007, K623)

OR

- Psychiatrist (SPEC=19): Any claim EXCEPT diagnostic codes 897-902, 904-906, or 909

**Best fitting ARIMA(p,d,q) models**

|  | Hospitalizations | Outpatient Visits | Lab Tests |
| --- | --- | --- | --- |
| CHF |  |  |  |
| Low user | AR(10,1,0) | AR(19,1,0) | AR(7,1,0) |
| High user | AR(2,1,0) | AR(6,1,0) | AR(5,1,0) |
| Mental illness |  |  |  |
| Low user | AR(4,1,0) | AR(9,1,0) | AR(4,1,0) |
| High user | AR(2,1,0) | AR(9,1,0) | AR(4,1,0) |
| COPD |  |  |  |
| Low user | AR(3,1,0) | AR(24,1,0) | AR(6,1,0) |
| High user | AR(5,1,0) | AR(6,1,0) | AR(4,1,0) |
| Diabetes |  |  |  |
| Low user | AR(6,1,0) | AR(6,1,0) | AR(4,1,0) |
| High user | AR(2,1,0) | AR(6,1,0) | AR(4,1,0) |

Figure 3. Weekly emergency department visits in high versus low virtual care users by medical condition, January 2018 to September 2020


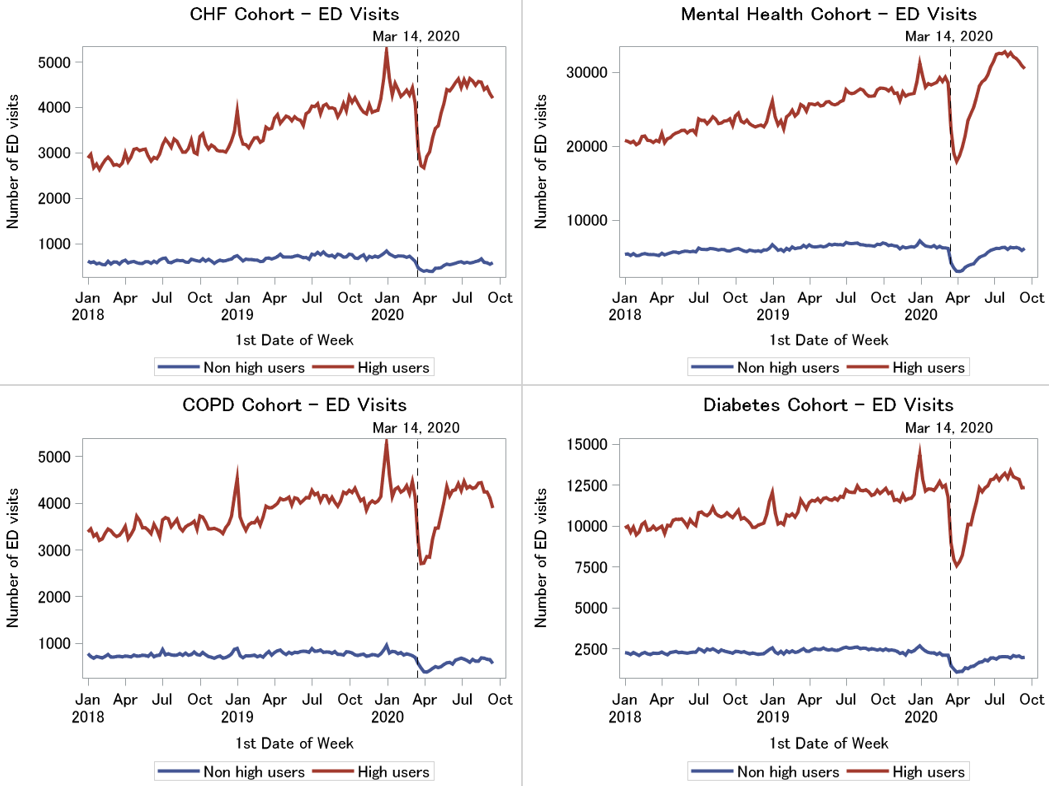


Figure 5: Weekly prescription claims in high versus low virtual care users aged 65+ by medical condition, January 2018 to September 2020

**
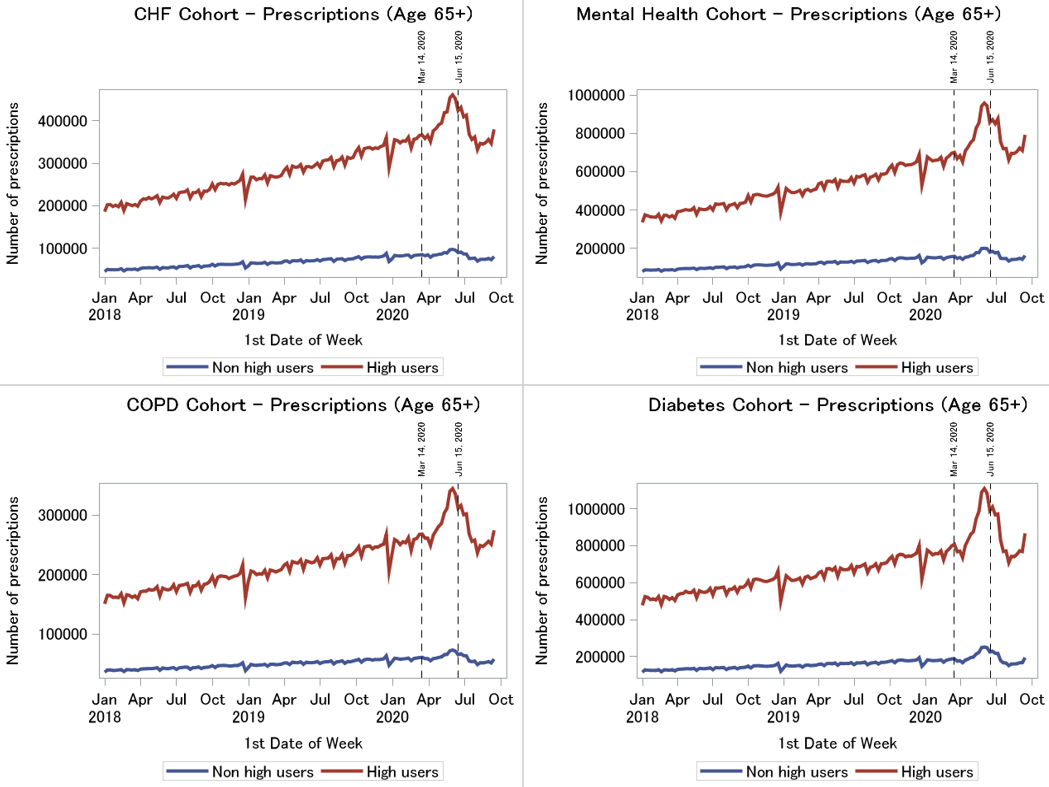
**

Figure 6: Mean cost per patient in low and high users of virtual care during the pandemic.


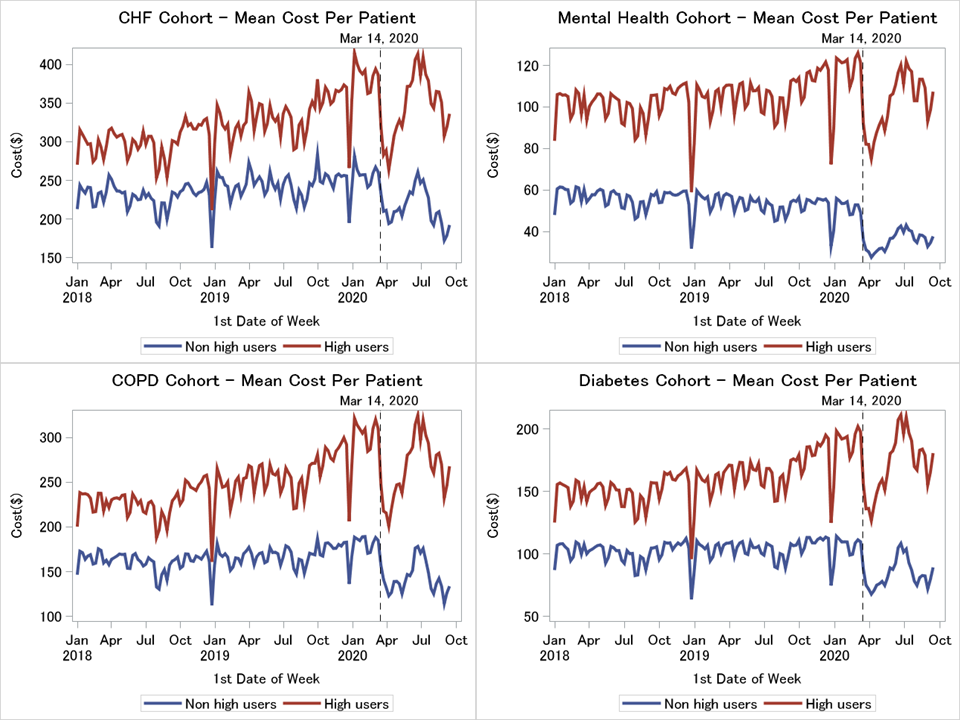

Supplement: S1 File — (DOCX) [file pone.0267218.s001.docx]
